# Supplementary material for: Cannabidiol Protects the Neonatal Mouse Heart from Hyperoxia-Induced Injury
Source: Int J Mol Sci. 2025 Dec 23;27(1):146. doi: 10.3390/ijms27010146 (PMC12785666; doi:10.3390/ijms27010146)
Supplement: Supplementary file 1 [file ijms-27-00146-s001.zip › Table S1 Sequences of Oligonucleotides.pdf]

Table S1. Sequences of oligonucleotides.

	Oligonucleotide Sequence 5′–3′	Accession No.
	18SrRNA	
forward	AAGTCGGAGGTTCGAAGA	NR_003278.3
reverse	GCGGGTCATGGGAATAAC	
	AIF	
forward	CCCAGAAGAGAAACAGAGAAG	NM_012019.3
reverse	ACCAATCAGCAGGAAAGG	
	Atg5	
forward	GCCTATATGTACTGCTTCATCC	NM_053069.6
reverse	AGTATGGTTCTGCTTCTCTTTC	
	Atg12	
forward	GCGAGAGCCTTCCAGAG	NM_026217.3
reverse	CCTTCAGCAGGATGTCAATTT	
	Casp3	
forward	TGACTGGAAAGCCGAAAC	NM_009810.3
reverse	GCAAGCCATCTCCTCATC	
	Col1a1	
forward	CACCAAACTCAGAAGATGTAGG	NM_007742.4
reverse	CAGGAGGACCAGGAAGT	
	CTGF	
forward	TGTGCACTGCCAAAGATG	NM_010217.2
reverse	AGGCAAGTGCATTGGTATTT	
	Cul7	
forward	TGCACATGTTGAGCAGTC	NM_025611.5
reverse	TGTTGGCTCAATGACAGAAG	
	CXCL1	
forward	AGACCATGGCTGGGATTCAC	NM_008176
reverse	GTGGCTATGACTTCGGTTTGG	
	CycD1	
forward	GCATCTACACTGACAACTCTATC	NM_007631.3
reverse	AGAGGAAGTGTTCGATGAAATC	
	CycD2	
forward	AGGACATCCAACCGTACA	NM_009829.3
reverse	AATTCATGGCCAGAGGAAAG	
	Gas2l3	
forward	CCAGGGAACTTTCCAATGAG	NM_001033331.2
reverse	CAAAGAGGTAAGTCTCGTCAAC	
	HIMF	
forward	CTGATGGTCCCAGTGAATAC	NM_020509.4
reverse	TCGTTACAGTGGAGGGATAG	
	Hmox1	
forward	CCGAGAATGCTGAGTTCAT	NM_010442.2
reverse	CGCTCTATCTCCTCTTCCA	
	IL1β	
forward	TGACGGACCCCAAAAGATGA	NM_008361
reverse	TGCTGCTGCGAGATTTGAAG	
	IL6	
forward	CACAAGTCGGAGGCTTAAT	NM_031168.2
reverse	GTGCATCATCGTTGTTCATAC	
	Lats2	
forward	GCAGATGCTTCAGGAGTT	NM_015771.2
reverse	CCAAGGCAGCTTCGATAC	

	Oligonucleotide Sequence 5′–3′	Accession No.
	Myh6	
forward	GACTGTCCGGAATGACAAC	NM_010856.4
reverse	CTCCAGAAGGTAGGTCTCTATG	
	Myh7	
forward	GGGAAGACTGTCAACACTAAG	NM_080728.3
reverse	GGGTTGGCTTGGATGATT	
	Nrf2	
forward	AGCACATCCAGACAGACA	NM_010902.5
reverse	GCTGCATACAGTCTTCAAAGTA	
	TEAD1	
forward	CAAACTCAGGACGGGAAAG	NM_009346.4
reverse	GTTACCTTCAGCTTGGAATGA	
	TGFβ	
forward	CTGAACCAAGGAGACGGAATAC	NM_011577.2
reverse	GGGCTGATCCCGTTGATTT	
	TNFα	
forward	ACCACGCTCTTCTGTCTACTGAACT	NM_001278601
reverse	TCTGGGCCATAGAACTGATGAGA	
	Tnni1	
forward	AGTGCCCTTCAGGACTT	NM_021467.5
reverse	CTTGATCTCTCTGGTGTTGTG	
	Tnni3	
forward	ATCTCCGCCTCCAGAAA	NM_009406.4
reverse	TGCCTCTCGTTCCATCT	
	YAP1	
forward	CCAGACGCTGATGAATTCTG	NM_009534.3
reverse	GGATGTGGTCTTGTTCTTATGG	

Abbreviations: 18S ribosomal RNA (18SrRNA); apoptosis-inducing factor (AIF); autophagy-related 5/12 (Atg5/12); caspase–3 (Casp3); collagen type I alpha 1 chain (Col1a1), connective tissue growth factor (CTGF); cullin 7 (Cul7); C-X-C motif chemokine ligand 1 (CXCL1); cyclin dependent kinase 1/2 (CycD1/2); GAS2-like protein 3 (Gas2l3); Hypoxia-induced mitogenic factor (HIMF); Heme oxygenase 1 (Hmox1); interleukin 1 beta/6 (IL1β/6); large tumor suppressor kinase 2 (Lats2); myosin, heavy polypeptide 6/ (Myh6/7); nuclear factor-erythroid 2-related factor 2 (Nrf2); TEA domain transcription factor 1 (Tead1); transforming growth factor, beta 1 (TGFβ); tumor necrosis factor alpha (TNFα); troponin 1/3 (Tnni1/3); Yes-associated protein 1 (YAP1).
