# Supplementary material for: Cannabidiol Protects the Neonatal Mouse Heart from Hyperoxia-Induced Injury
Source: Int J Mol Sci. 2025 Dec 23;27(1):146. doi: 10.3390/ijms27010146 (PMC12785666; doi:10.3390/ijms27010146)
Supplement: Supplementary file 1 [file ijms-27-00146-s001.zip › Figures S1,S2,S3.pdf]

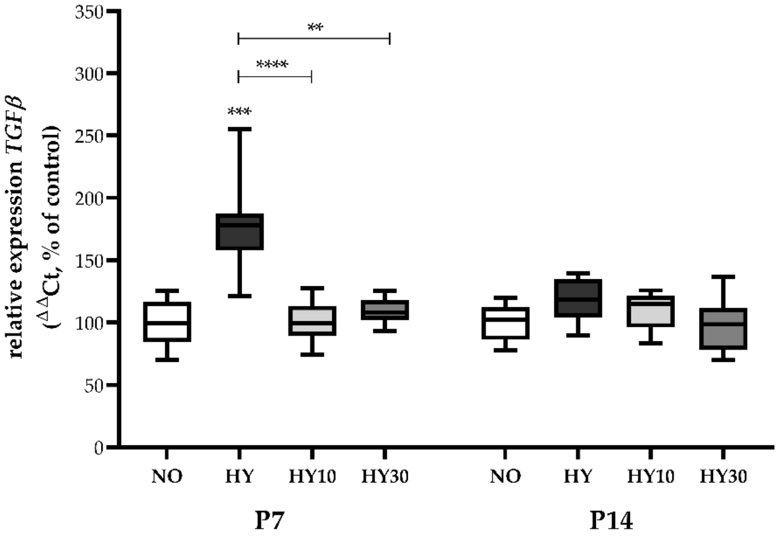

Figure S1. Quantification of fibroblast-associated transcripts for *TGFβ* of whole hearts for P7 after 2 days oxygen exposure and recovery until P14 using qPCR. Data are normalized to the level of mouse pups exposed to normoxia at each time point (control 100%, NO, 21 % O_2, white bars) with verum groups hyperoxia (HY, 80 % O_2, black), hyperoxia with CBD 10 mg/kg (HY10, light grey), and hyperoxia with CBD 30 mg/kg (HY30, dark grey). Data are presented as Box-Whisker-Plots with n=12 per group. ** p<0.01, *** p<0.001, **** p<0.0001 (ANOVA).

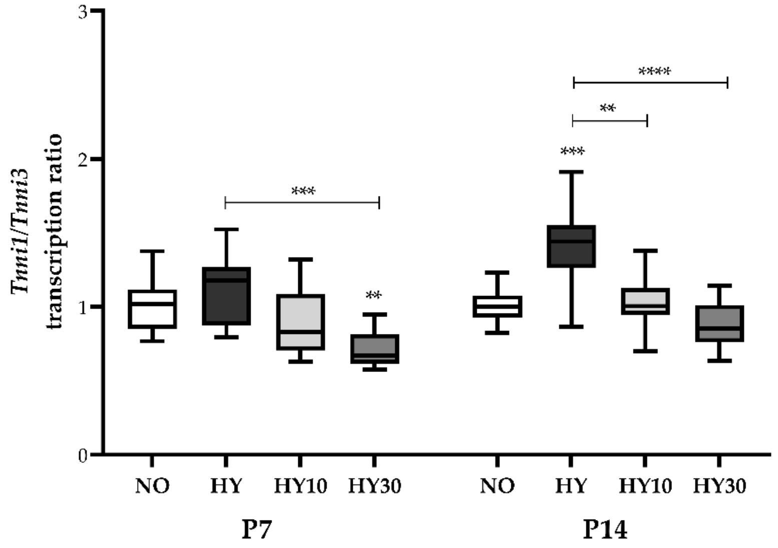

Figure S2. Quantification of cardiac remodeling transcripts for *Tnni1*/*Tnni3 ratio* of whole hearts for P7 after 2 days oxygen exposure and recovery until P14 using qPCR. Data are normalized to the level of mouse pups exposed to normoxia at each time point (control 100%, NO, 21 % O_2, white bars) with verum groups hyperoxia (HY, 80 % O_2, black), hyperoxia with CBD 10 mg/kg (HY10, light grey), and hyperoxia with CBD 30 mg/kg (HY30, dark grey). Data are presented as Box-Whisker-Plots with n=12 per group. ** p<0.01, *** p<0.001, **** p<0.0001 (ANOVA; Brown-Forsythe).

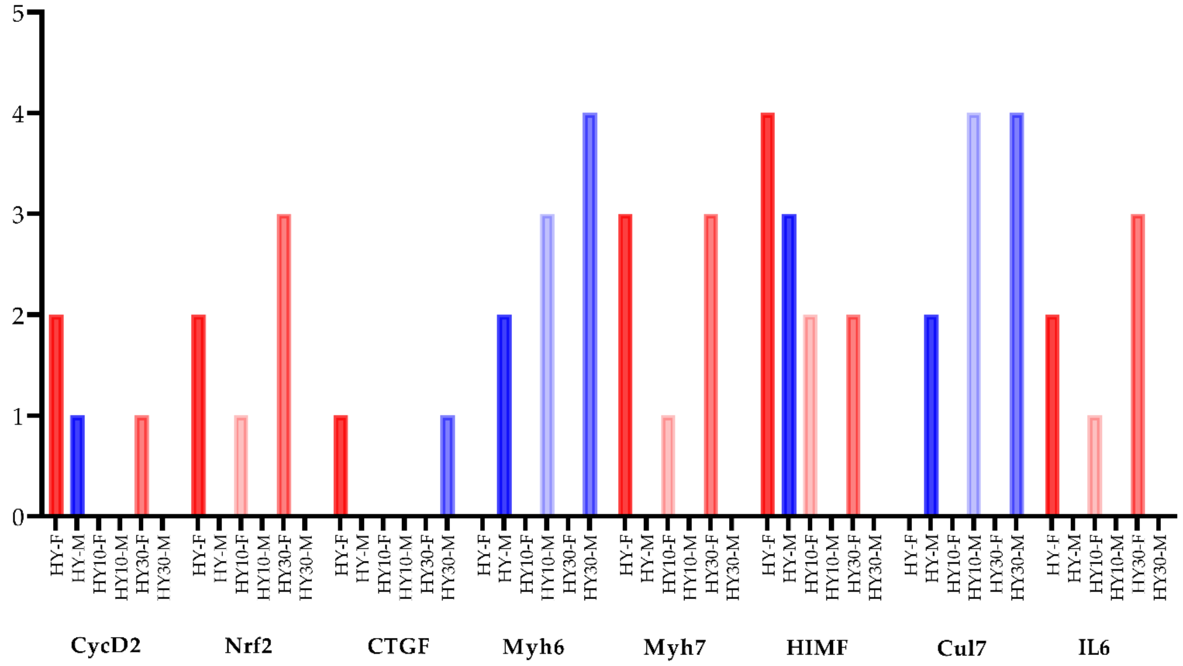

Figure S3. Effect size of gene expression in female (F, red) and male (M, blue) samples exposed to hyperoxia (HY, 80% O_2), with pretreatment 10 mg/kg BW (HY10, bright red/blue), or pretreatment 30 mg/kg BW (HY30, medium red/blue). The grouped bar graph displays effect size levels for each gene under the three experimental conditions.
